# Cruel optimism, affective governmentality and frontline poverty governance: ‘You can promise the world’

**DOI:** 10.1111/1468-4446.13144

**Published:** 2024-09-07

**Authors:** Edith England

**Affiliations:** ^1^ School of Education and Social Policy Cardiff Metropolitan University Cardiff UK

**Keywords:** affective governmentality, cruel optimism, homelessness, neoliberal paternalism, performativity

## Abstract

Cruel Optimism’ (Berlant, 2011) sustains neoliberalism by promising freedom and autonomy through adherence to and performance of competitive behaviours. As Brown (2003) observes, neoliberalism is a discourse which operates, not through repression or restriction, but through promising self‐fulfilment and happiness. The role of emotion‐management in poverty governance has been widely acknowledged. However, this has focused on cultivation of population‐level punitive, negative emotions (such as shame, stigma, or resentment). It is widely acknowledged that welfare provision has been specifically targeted by neoliberal discourse, justifying intensifying interventions aimed at reshaping the subjectivities and aspirations of poor and marginalised individuals and households to serve the needs of deregulated markets. However, little attention has been paid to the importance of positive, hopeful emotion management in legitimising and effecting co‐operation. Drawing on interviews with 54 workers in the Welsh homelessness system, I argue that workers systematically create and sustain optimism in their clients as a mechanism to enable them to survive within an increasingly hostile housing system, as part of a deliberate, if reluctant, strategy to cultivate empowered, ‘ethical’ welfare selfhood against a backdrop of citizen abandonment. A three‐stage approach deployed by workers includes (1) destabilisation of expectations of state help (2) re‐orientation, through cultivation of belief in neoliberal promise (3) development of maintenance strategies. Improving applicant capacity to perform neoliberal welfare citizenship was perceived as an urgent, moral and pragmatic necessity, and justified by care logics. I demonstrate how this extends not only our understanding of welfare implementation, but also shows how positive emotion‐management generally, and Berlant's Cruel Optimism specifically, can be used to understand the practicalities of welfare governance.

## 1 INTRODUCTION

Neoliberalism – here understood, following Brown (2003), as a discourse of privileged free‐market expansion, deregulation and a reduction in government spending, which operates as *‘a distinctive mode of reason…production of subjects, a ‘conduct of conduct’ and a scheme of valuation’* (Brown, 2003, p. 5) ‐ has been widely recognised as transforming the relationship between the state and those in receipt of welfare support from a paternalistic moralism rooted in an understanding of welfare subjects as incapable, to a tutelary conditionality where assistance becomes dependent upon co‐operation with state mandates (Clarke, 2005; Cruikshank, 1999; Flint, 2019). However, this literature focuses almost exclusively on social control as punitive or repressive, overlooking a more fundamental aspect of neoliberalism – its ability to operate as promissory potential, inspiring, enrolling, and engendering subjectivity through desire and imagination (Brown, 2003; J. Tronto, 2017).

Frontline welfare workers operate in challenging and changing structural conditions. Compared to those receiving services, they are often relatively powerful, able to make critical decisions (such as whether an individual receives access to food, shelter or financial support, or whether their children are removed from the family home). Yet these decisions are often made under substantial pressures, amid competing demands of time and resources, and increasingly often subject to challenge and scrutiny England, (2022); Soss et al., 2011). They are also increasingly disempowered due to ongoing defunding and retrenchment of the welfare state (INCITE!. et al., 2007; Schram, 2019). Although historically, frontline workers involved in welfare administration have been understood as relatively emotionally detached from their clients (Alden, 2015; Gill, 2016), there is also increasing evidence that they deploy considerable emotional and affective labour as part of their roles, especially to compensate for the impact upon clients of welfare abandonment and responsibilisation (England, 2022; Penz et al., 2017; Silver, 2010; Soss et al., 2011). However, widespread systemic resource shortage creates a moral quandary, wherein workers within the system know that their clients are disadvantaged and yet have very limited options available to prepare them to survive this system. Explorations of how this dilemma is resolved has, to date, primarily focused upon use of negative and revanchist interventions designed to cultivate compliance based upon a sense of fear and punishment. In this paper, I explore another approach. Extending Berlant's theory of ‘Cruel Optimism’ (Berlant, 2011), wherein desire and hope for a better future are used to engender and sustain compliance, I explore how welfare clients can and are motivated to comply, through overt and deliberate strategies by frontline workers, by the belief that this system, despite being highly flawed, is still better than the alternatives. This reflects ongoing work on neoliberal citizenship reformulation, which has long recognised that neoliberalism operates primarily not as a punitive, negative force but through promising freedom and power (Brown, 2003; Young, 2000).

I draw upon interviews with 54 workers in the Welsh homelessness system to consider how frontline homelessness workers in the Welsh system enable their clients to accept, adapt and survive within neoliberal reform. The Welsh homelessness system offers an especially useful location to study the operation of neoliberal affective governance: rates of sanctions are very low, suggesting that power is largely discursive in nature (AUTHOR REMOVED, 2023). As such, the system presents an unusual situation where coercion is largely absent and emotional and affective power has elevated importance. I argue that workers in the Welsh homelessness system systematically create and sustain optimism in their clients as a mechanism to enable them to survive within an increasingly hostile housing system, as part of a deliberate, if reluctant, strategy aimed at cultivating empowered, ‘ethical’ welfare selfhood against a backdrop of citizen abandonment. Using Berlant's theoretical approach enables a three‐stage approach deployed by workers to become evident, including (1) destabilisation of expectations of state help (2) re‐orientation, through cultivation of belief in neoliberal promise (3) development of maintenance strategies. Existing studies have largely explored Berlant's theory as a static discourse. This study demonstrates that Cruel Optimism is not simply a prescriptive, top‐down, discourse but rather is a negotiated, pragmatic set of strategies, reflecting Foucault's observation that subjectification must be tailored and specific to be effective (Foucault, 2020). This paper makes two further important contributions. First, it demonstrates the utility of hope as an analytic to understand the pervasive spread of neoliberal rationalities among the workplace practices of those at the frontlines of welfare governance. Second, it extends Berlant's approach by exploring its temporal after‐life, including through applying their largely‐theoretical framework to empirical data. It also advances existing research (for instance those by (Alden, 2015; England, 2023, England and Henley, 2024; Bretherton et al., 2013; Glinsner et al., 2019; Penz et al., 2017; Silver, 2010) which illuminate the affective complexity of frontline work, by highlighting the emotional labour involved in the repeated social and moral engagement and decision‐making which comprises the role.

The rest of the paper is structured as follows. First, I discuss the increasingly complex affective lives of frontline workers and explore the utility of Berlant's work for understanding neoliberal system change. I then contextualise the study amid the shift to a neoliberal paternalistic approach within Welsh homelessness provision, and discuss the methodological approach taken. Next, I discuss findings, before concluding with a discussion of the significance of the study's findings in complicating previous simplistic understandings of the subjectivities and motivations of frontline workers.

### 1.1 Affective governance and the neoliberal turn

Brown (2015) offers a critical framework for understanding the rationalities of modern governance, proposing neoliberalism to primarily operate as a political, discursive, rationality, through which the market becomes understood as the normative arena through which societal values, interpersonal relations and expectations are structured. Following Foucault, she proposes this discourse to operate dynamically and dialectically, actively creating individual subjectivities through apparent freedom and opportunities to participate in power, more than restriction and punishment. Brown further argues that this operates within a wider economic context in which citizens must take individual responsibility for themselves due to substantially reduced state provision, with citizenship itself becoming contingent upon performances of idealised, aspirational economic rationality. Clarke (2005) has traced the implications of a discourse of personal responsibility within British welfare provision, arguing that it forms a key component of modern poverty governance, while Joseph (2013) has demonstrated the specific importance of ‘resilience’ as personal responsibilisation for future risk, as a discursive mandate within poverty governance. Schram et al. (2013) have argued that, within welfare systems, this takes a specific form – which they term ‘neoliberal paternalism’ – which is distinctive for attempting to create, shape and commodify poverty through both a restructuring of administrative‐bureaucratic state apparatus, and intensification of emotional‐authoritative pedagogical interventions. As (Cruikshank, 1999) notes, although punitive and moralistic systems of welfare governance are not new, the last 40 years have seen a shift toward embedding these within a distinct wider moralistic‐discursive strategy where poor citizens are trained to both desire and internalise the wider aims and needs of neoliberalism.

The role of affect and emotion as part of poverty governance has increasingly been of critical importance in understanding the operation of the revised welfare system. This work has increasingly considered the interactions at the frontlines of governance, especially between workers and applicants. Sauer and Penz (2017) use the concept of ‘affective governmentality’ to explore the importance of emotionality to governance.

Crucially, and following Brown's (2015) focus on neoliberalism as a discourse that operates through creating subjectivities rather than punishment or constraint, ‘affective governmentality’ operates primarily through creating emotional bonds between state actors (including frontline workers) and their clients, with the aim of persuading the latter to comply in order to fulfil implicit social‐emotional obligations.

A related strand of literature has also considered how emotion is used in a context of devolved authority to responsibilise welfare claimants for state withdrawal, for instance by sharing privileged ‘backstage’ (Goffman, 1978) fear‐inducing information about state withdrawal to motivate applicants to act independently. With reference to the Welsh homelessness system, England, 2023 has shown that frontline workers strategically and selectively share information about risks of non‐compliance with their clients in order to convey to them the urgency and desperation of their situation and motivate them to take personal responsibility, acting not through revanchism but because, in a resource‐constrained system, their alternatives are highly limited. In this setting, the relationality, boundaries and distance between welfare citizens and those administering the system are liminal and complex. Penz et al. (2017), for instance, explores how authority in this situation operates through alternating and nuanced narratives, creating what might be termed a patchwork emotional performance which enables tailored, specific and so effective governance to operate through the actions of frontline workers (see also England and Henley, 2024; England, 2024).

This‐form of affective governance is especially powerful because it enables social control to occur indirectly and instinctively, with relevance for those claiming welfare. For instance, abjectification (Allen et al., 2014; Tyler, 2013) creates certain individuals as *defacto* objects of disgust, promoting both fear and aversion to the approaches associated with them and hence operating as a moral deterrent. Tyler (2013) sees this creation of an othered and stigmatised class of welfare citizens as critical to effective poverty governance, arguing that this ’governmental technology of division and dehumanisation’ (Tyler, 2020, p. 7) has been used to legitimise both anti‐welfare sentiment and anti‐migrant sentiment. She draws attention to the use of the rhetoric of class‐based disgust to establish the fundamental otherness of stigmatised groups, particularly welfare subjects (Tyler, 2013, 2020, see also K. Allen et al., 2014; Jensen & Tyler, 2015). Strong (2021) suggests that similar mechanisms are used to deter people from using foodbanks, and Allen et al. (2014) have traced how these narratives produce gendered justifications of welfare exclusion when applied to women, especially mothers, claiming welfare (see also (England and Henley, 2024; Cruikshank, 1999).

Scholarly exploration of affect as deployed by state agents in the welfare system has, however, almost entirely focused upon the deliberate use of negative emotions as deterrents. Given Brown's (2015) argument that neoliberalism is successful precisely because its primary mode of operation is not punitive or deterrent, but rather enticing and enrolling, succeeding through offering hope and desire, it would seem critical to consider whether, and how, deliberate and strategic cultivation of positive, hopeful affective states might form part of poverty governance. The possibility that welfare subjects might be governed through optimism and hope has been notably absent from studies of affective governmentality.

### 1.2 Neoliberalism, cruel optimism and the logics of poverty governance

Berlant (2011) argues that the promissory hope offered by neoliberalism is an affective state through which a wider attachment to the conditions and limitations of neoliberal existence becomes sustainable. This ‘cruel optimism’ (Berlant, 2011), reliant upon a discourse of ‘toxic positivity’, tethers the individual to an existence where they cannot flourish, by creating a psychological dependency upon the possibility of utopian futurism. To Berlant it is this affective engagement, comprised of instinct, feeling and desire, rather than a logical, rational appraisal of benefits and risks, which best explains *sustained* alignment with a system which frequently disappoints. Once affective attachments are formed they are strong and transcend rationalities. They highlight the importance of emotional attachment to institutional norms and idealised subjectivities – the ‘good mother’, the ‘deserving welfare subject’ or the ‘hard working family’ – as a mechanism for ensuring compliance, arguing that people have a deep desire to be accepted and recognised according to neoliberal logics, even where this causes personal, individual disadvantage.

Cruel Optimism is useful in explaining why individuals accept poor treatment and neglect of their needs, without losing belief in the logic or value of neoliberal social structures (Brown, 2003; Skeggs, 2014; Young, 2000). It provides a frame to understand how apparent hope – notably through the alignment of choice with freedom (Brown, 2003; Young, 2000) – can operate through embodied materialities as a tool of symbolic violence upon those dependent on increasingly atrophied state support. It explores how affective, cultural discourses can produce compliant citizens even in the absence of the diffuse threat characterising Foucauldian panopticism (1979), Deleuzean apparent, yet constrained, choice (2017), or interpolation (Butler, 1997), suggesting that the suffering *caused* by capitalist failure to address social inequalities can be understood as an opportunity for competitive self‐development and so a vector to increased future opportunities. This reframing helps to explain how individuals continue to comply with harsh and often ineffective demands made upon them, for instance by neoliberal welfare systems, despite a clear contradiction between their actual experience of the system and their lived experience.

Berlant conceptualises Cruel Optimism as a process with distinct stages. Here I focus upon three: disruption, re‐orientation and maintenance. Disruption involves an *affective event* (Berlant, 2011, p. 846) which challenges the individual's stable worldview. This creates instability, panic and disorientation which is then conducive to encouraging individuals to accept disadvantageous change as a necessary, pragmatic, survival mechanism, or ‘crisis ordinariness’ (Berlant, 2011, p. 10). Re‐orientation involves exposure to cultural discourses which showcase the possibilities of a promised, idealised future (Berlant, 2011). Finally, in the maintenance stage, individuals learn to live in crisis *despite* the clear contradictions between what has been promised and what can plausibly be achieved. While the first two phases (destabilisation and recouperation) share conceptual similarities with processes within Foucauldian governmentality (McKee, 2009), Berlant's attention to this longer term maintenance phase is distinct, and important in accounting for how individuals and societies live in crisis over an indefinite period without ceasing to believe in the fundamental logics of neoliberalism. They explore different ways to characterise this phase, including *crisis ordinariness* and *slow death,* representing a normalised failure as a routine, mundane, unremarkable mode of existence. Its very ordinariness stabilises previously remarkable, even abhorrent, rationalities as normal governing logics of daily life. It is sustained because the act of desire for this attachment upholds the subject's sense of self, by providing a cohesive narrative which centres them as autonomous, valid and capable individuals connected to, and valued by, wider society. It therefore offers a structure for living in a way which promises a good, free and successful life, narrating *‘what it means to keep on living and look forward to being in the world.’* (Berlant, 2011, p. 20).

As discussed in the previous section, neoliberalism renders welfare citizenship a site where people are blamed and stigmatised, rendering them vulnerable and desperate with little choice but to engage. Previous studies of frontline welfare interactions have largely considered how coercion and both positive and negative interpersonal regard are used, especially through casework, to instigate and sustain behavioural change. it has largely been considered as a way to understand how individuals resolve contradictions between promises and reality across a range of areas, including education (Bone, 2021; Lipton, 2020; Moore & Clarke, 2016), marginalisation (Brunila et al., 2021; Kettle & Jackson, 2017; Runswick‐Cole & Goodley, 2015) and gender (Cappellini et al., 2019). However, as I demonstrate in the subsequent sections, when considered within the wider context of neoliberal welfare reform, it can also be understood as an explicit and routine hegemonic tool which offers insights into the contradictions at the frontlines of neoliberal welfare governance.

## 2 METHODS

### 2.1 Welsh homelessness services

The nature and scope of homelessness provision in the UK has long been contentious. While the UK has, for the past 40 years, incorporated a statutory right to access to state housing for those recognised as ‘vulnerable’, for a majority, provision has been minimal. The wider context of British housing has been repeatedly implicated in high rates of homelessness and long term housing precarity. Owner‐occupation is declining rapidly in the UK reflecting increasing financialisation of housing (England, 2023). The British private rented sector, often characterised as a short‐term option for those seeking housing flexibility, is increasingly providing long term accommodation for those priced out of owner‐occupation. The sector is deregulated in terms of rents and quality, and security is minimal, with most tenants entitled to a one‐ or two‐month notice period. Tenancy security is further compromised by ongoing austerity‐based cuts which target assistance with housing payments for those in rented accommodation. Consequently, evictions from the private rented sector are a significant causal factor in households becoming homeless (England, 2023).

The last five years have seen several key changes within the British homelessness system, largely bringing statutory homelessness provision into alignment with welfare systems. The Housing (Wales) Act 2014 (and subsequently the English Homelessness Reduction Act, 2017) has been widely regarded as a significant development in British, and international, homelessness law, substantially broadening the range of those entitled to homelessness assistance and introducing a focus on prevention of homelessness. However, a less discussed aspect of the new law is the introduction of welfare conditionality. Applicants must now demonstrate that they are actively attempting to resolve their own homelessness, or risk having assistance terminated (see AUTHOR REMOVED, 2023). The implications of assistance being terminated are far reaching, translating to exclusion from (relatively more secure and better quality) state housing and lack of access to financial help to secure and retain private rented accommodation (e.g., deposit schemes and assistance with rent). Engagement with the local authority centres upon overt and visible attempts to secure private accommodation, echoing the ubiquitous workfare approach discussed above (Mackie et al., 2017). Occurring in the wider context of rapidly rising rents, reduced state support with housing costs (particularly targeted at those with dependent children), and substantial stock reductions in social housing (MacLeavy, 2011; Morris, 2016; Powell, 2015), this approach has elsewhere been critiqued for placing unrealistic and unattainable demands upon both highly precarious applicants and staff (England, 2022, 2023).

### 2.2 The study

I draw on data from a doctoral study considering the enactment of the Housing (Wales) Act 2014 from the perspectives of both frontline workers and applicants themselves. I re‐analyse extended, open‐ended interviews with 54 frontline workers to understand how normative interactions during a homelessness application can be symptomatic of a systemic cruel optimism. All interviews were conducted between September 2018 and January 2020, and were recorded and transcribed by myself. Ethical approval for the study was granted by the School of REMOVED, REMOVED University Ethics Board.

Workers were asked to reflect upon their normative practices over the lifetime of a homelessness case. All had responsibility for individual homelessness decisions in a casework role, with 30 directly employed by the local authority in a role which explicitly involved decision‐making over applications (‘decision‐makers’), and a further 24 in support roles affording them functional decision‐making power within cases, especially whether behavioural conditions had been complied with. Reflecting the elevated importance of the ‘shadow state’ within Welsh homelessness law (England, 2022; Drakeford, 2012; INCITE!. et al., 2007), around half this group were employed by organisations funded by, and usually co‐located within, the local authority (the remainder were directly employed by the local authority). Nearly two thirds of the total group were female, with the remainder male or non‐binary. Reflecting the relatively small geographic area from which participants were recruited, and their status as low‐waged, often precarious, staff, detailed demographic information about specific staff is not shared, to avoid risk of identification.

Most participants were recruited from their workplace, although a small number made contact following applicant social media recruitment. Interviews were semi‐structured and lasted around an hour on average, (ranging from 30 min to around 2 ½ hours). They were audio‐recorded and transcribed verbatim including untimed pauses, hesitations, repetition, emphasis and interruptions, and to reflect dialect variations. In reproducing analysed extracts, some concatenation/textual deletion is occasionally used to improve clarity: this is noted by ‘…’ where it occurs.

#### 2.2.1 Analysis

The data corpus analysed for this paper totalled over 100 h of transcribed interviews. These were iteratively coded according to Braun and Clarke's (2006) six analysis phases (familiarisation, initial coding, initial theme allocation, theme development, theme refining and narrative congruence). This thematic approach enabled a focus upon the hidden, less visible aspects of the data – going ‘*beyond the semantic content of the data’* to ‘*identify the underlying ideas, assumptions and conceptualisations – and ideologies‐ that are theorised as sharing or informing the semantic content of the data.*’ (Braun & Clarke, 2006, p. 84). This was informed by a Critical Discourse Analysis approach, offering a tool to understand the perspectives of participants in the context of shifting, competing and situated social power (Fairclough, 2005). It is useful in exploring how ideas operate across different organizational contexts to create and uphold structures which justify exclusion (Wodak & Meyer, 2015). This approach recognises discourse as productive, operating dialectically with personal and organizational practices, and contributing to normalization and integration of new ways of working (Fairclough, 2005). Yet it also attends to the radical, resistive and pragmatic power of discourse and so is especially useful in making visible social experiences which have previously been overlooked, erased or dismissed. Used in this study, it operates to centre the social understandings within interactions at the frontlines of homelessness governance, and especially to make visible the logics through which workers interpreted, justified and maintained their social location.

This study used Critical Discourse analysis to explore how workers understood their role, including their interactions with applicants. It was especially useful in identifying different layers of discourse, for instance where workers saw themselves as producing a subversive or resistive viewpoint – one contrary to the normative and socially dominant ‘*party line’* as one decision‐maker put it. As such, it was helpful in exploring how workers saw themselves and their role *within* a wider context in which they saw themselves as both powerful and powerless, and how this justified and explained their interactions with applicants.

### 2.3 Destabilisation: *‘If they're not scared…I don't think they get it, the safety net's gone’*

Following Berlant's (2011) proposal that cultivating a sense of personal inadequacy is critical to engendering a willingness to change (Brown, 2003; Young, 2000), I argue that workers saw their role as to destabilise and unsettle applicants' expectations of state help. Creating fear and urgency (rather than anger), which leads to hope (specifically hope in opportunities available in the private rented sector)_enabled failure to be reframed as progress, necessary to move them to a position of acceptance of their own need to take responsibility for resolving their own homelessness. Workers regretted the lack of state support available to applicants, but also emphasised the importance of pragmatism through responsibilisation, which, through positioning the present as a risk to the future, because current practices did not enable applicants to survive neoliberal abandonment.

They explained that state withdrawal meant that they *needed* applicants to become motivated by the strong, self‐preservation‐based, emotions engendered by raising their awareness of the loss of forms of support they had previously taken for granted. Workers tended to see themselves as managing messages around an incoming crisis to motivate applicants to act. As one worker put it, ‘*I want them to be scared. Yes I do. Because if they're not scared, without that fire…I don't think they get it, the safety net's gone.’*

They encouraged applicants to see their current mode of being as *dangerous* in its unsustainability. Understanding themselves as pragmatic, with insider system knowledge, they saw themselves as having a responsibility to responsibility to persuade applicants to change their understanding and approach. They widely saw applicants as naïve and incapable of recognising the threat they were now under, making this ‘*wake up call’*, as several workers described: a protective act of care and kindness which could, by forcing a state of individual responsibilisation, avert future hardship. As one hostel worker explained, *‘I need them to get their head around it…you know if you keep doing the same old thing over and over, it's not how it once was. There's no one there to catch you when you fall, those days are long gone.’*

Reflecting Berlant's (2011) observation that crisis cultivation is a primarily *affective* state, workers saw applicants' emotional situations as critical locations of interventions. Affective responses to crisis, such as fear and panic, were deliberately cultivated as tools to engender visceral awareness of the increasing risks of not accepting responsibilisation. They saw the greatest threat to applicants being complacency – a widespread lack of awareness of the crisis. Normalising a withdrawn, conditionalized homelessness system, and asserting it to be true and rational, was also evident in the language used by workers to describe the new approach. They described practices such as performing a ‘*reality check’*, *‘making it real’*, with pragmatic explanations ‐ *‘just the way of it.’* Meanwhile, belief in the capacity of the welfare state to provide for them, reflecting a nostalgic belief in a ‘golden age’ of housing provision, were represented as both naïve and dangerous‐ as being in *‘la la land with no idea of what's going to hit them, we're in for a tricky time….and that's just me being real, you know’* (decision‐maker). These practices of rationalisation‐justification constituted the new system as *a* ‘*field of knowledge…constitute[ing] at the same time power relations.’* (Foucault, 1979, p. 27).

A related strategy used by workers to normalise crisis was to underline the futility of challenge. Workers frequently acknowledged that the system was unfair. However, they did so in such a way as to model the impossibility of escape from the crisis except through performing compliance. They described how they managed applicants' emotional reaction to unfairness and marginalisation – for instance anger and frustration – to frame theme to applicants as temporary, unproductive, and self‐limiting obstacles to overcoming static, self‐indulgent, failure. Applicants were encouraged to see their anger at structural exclusion as a childish phase – *‘you can stamp and shout, rage all you like, but that's not going to get you a house so I say, let it out and then come back and we'll try again*.’ (decision‐maker). A worker in a busy hostel articulated how she justified her strategic approach in a weary, cynical way, articulating complaint and resistance as not only pointless but, in interrupting the process of searching for housing, potentially dangerous.

It’s the way of it now, it’s how it works, but we get ‘human rights’. I say to them, I say listen you can complain all you like. Who to? No idea, to be honest. Write to your MP, we get that a lot. I don’t know who to! but knock yourself out. When you’re done with that, I’ll be here waiting.

Workers therefore saw curating a sense of crisis urgency as an essential tool in engendering receptivity to welfare abandonment. They saw their role as to communicate a need for change to applicants, believing that they were often incapable of recognising the urgency of doing so for themselves. As I show in the next section, this enabled new framings of the relationship between impoverished citizens and the state to then be proposed.

### 2.4 Re‐orientation and the power of imagination: *‘They need to be aiming somewhere’*

Berlant (2011) proposes that cultivating desire, through reference to hope and possibility, is a governance technology which operates through responsibilising citizens for their own marginalisation, by promoting success as possible if they are willing to behave as correct neoliberal citizens (Berlant, 2011; Lynch et al., 2020). Workers saw themselves as actively harnessing imagination as part of a strategy to cultivate optimistic attachments (Berlant, 2011), drawing upon possibility – encouraging applicants to frame things in terms of what could be, through a structured programme involving curating the idea that desire is the correct route to success. Without motivation to change, they proposed, applicants would simply remain homeless in the future. They saw hope as a tool through which change became possible, by providing both motivation and direction: a precious commodity with a use value, with optimism a resource to engender openness and receptivity (Pykett, 2010; Rose & Miller, 2010). A worker in a family hostel articulated the importance of desire: *‘If they've got no ambition, there's nothing to work with…they need be aiming somewhere.’*

Workers had well‐developed strategies for honing applicant motivation. They reported asking applicants to describe housing aspirations, scrutinising within a neoliberal, market‐based framework. Willingness to strive for private rented housing, compromise and defer present needs were celebrated. Expecting ongoing state support was regarded as short‐termist, infantile and unfeasible. Expecting little, taking responsibility and performing gratitude meant applicants were understood as deserving (Jensen & Tyler, 2015). One worker explained how she felt it to be important to develop, in applicants, neoliberal ideas of self‐sacrifice and delayed gratification. She used successive choices, and so underlined, to applicants, the impossibility – and implicit arrogance ‐ of, as she put it ‘*thinking the rules don't apply, thinking oh well I'll be having it all’*. She continued:

…you have to make sacrifices…you have to be willing to compromise. You want a three bedroom house in [expensive area]. With a garden for your kids! And a swing! [laughs]. It’s unlikely. [laughs]. So then we get into, what’s the most important to you? Is it the garden with the swing, or is it having three bedrooms? Is it [area]? … It’s about compromise.

Another strategy to cultivate imaginative optimistic attachment was visualisation. Workers described talking applicants through fantasies of private rented sector success, using an affective dimension to strengthen motivation and engagement by creating tangibility. They saw this as important in asserting the feasibility of securing private rented sector accommodation, including for this specific applicant. One family hostel worker explained:

I’ll say to them, you’re opening your front door – just hold that in your head now…how does that feel? It sounds…Disney…yeah but I’m about, I’m making it real for them. You can do it, yes you can.

Workers saw developing a belief in self‐ownership of their homelessness among applicants as a critical investment, framing it as an act of care: an investment in the applicant's longer term survival which they performed not only as a housing worker but as a connected human being (England, 2022). This re‐orientation was reported as a highly satisfying, affectively motivating aspect of their role – a *‘key moment… the penny drops, it's down to me, I can do it…’* (outreach worker). Another worker, a decision‐maker in a busy city centre office, celebrated the relief of *‘the breakthrough moment…you see it in their eyes…that's when I think you're going to be ok love.’*

Workers often spent considerable periods of time trying to cultivate applicant optimistic attachment to private rented accommodation. A decision‐maker described this process over the period of around two years with one client. Her client was cyclically homeless, with repeated periods of incarceration, and felt strongly that he should be allocated state accommodation. When her client grew frustrated at being told he had to look for private rented accommodation, the worker took the brunt of his anger: *‘He wasn't having none of it. ‘Find me a flat!’ Effing and blinding and I was the [only] one could work with him.’* She saw instilling both an awareness of, and desire for, private rented housing as a moral obligation; to fail to do so, she believed, increased his risk of homelessness considerably. She explained how she carefully reframed the precarity and unaffordability of the private rented sector in terms of choice and freedom, understanding her role as to help him value the search for poor quality, insecure accommodation as part of a progression toward more stable housing. ‘*I said to him, private rented, ball's in your court, world's your oyster…you don't try, you don't get, do you?’.* Hope and optimism then became retooled as opportunities for change, shaping behaviour and providing ongoing motivation for compliance.

It’s that moment…that’s a little bit of hope right there…a leap of faith…Ok let’s work with that, let’s use that, yeah, let’s turn that into something that counts…if there’s one thing I’ve learnt it’s this: you don’t waste hope.

Workers prioritised development of faith in the feasibility of finding accommodation within the private rented market. Yet, as I show in the next section, the reality of well‐documented difficulties with finding private rented accommodation, especially for those experiencing homelessness, threatened sustained belief in the system.

### 2.5 Maintenance through pragmatism: *’You can't let them wallow’*

Neoliberalism must sustain compliance despite inherent and apparent contradictions between promise and reality. Berlant (2011) argues that this is achieved through a negotiated, often precarious, process of sense‐making – ‘*stories about navigating what's overwhelming.’* (Berlant, 2011, p. 10), through which exceptional, jarring changes become reframed as logical, even desirable, sequiturs of current social arrangements. Ongoing *‘crisis ordinariness’* (Berlant, 2011, p. 10) sustains long term faith in neoliberal promise despite clear evidence that the strategies they are encouraged to adopt are unlikely to be successful – *‘as life goes on, habituation does too.’* (Berlant, 2011, p. 81). In this section I argue that frontline workers were invested in the use of long term, emotionally entangled, maintenance strategies which re‐narrativised failure as a stepping‐stone to success. In this stage, applicants were mentored in how to move from the state of (proposed) self‐limiting anger typifying the disorientated, destabilisation phase, and into a longer term, more managed, desperation where the focus of their frustration was turned inward to motivate them to continue to search for accommodation.

To sustain applicants through successive, inevitable, failures during their attempts to secure housing, workers renarrativised these struggles as progress. In this way, setbacks became incorporated into a neoliberal framework of ongoing progress – not as evidence of the impossibility of the task, but as a demonstration that, although success was complex and difficult, applicants were increasing their chance of success. This was often framed in terms of learning, with struggles and obstacles operating as opportunities to improve understanding and develop strategies. One decision‐maker explained that, while recognising the challenges of the task – *‘What we're asking them to do, it's not easy, I don't believe in sugarcoating it’* it was important to him to frame this as part of an *overall* progress toward successfully securing permanent housing. He discussed this in the context of applicants learning strategies to quickly secure properties once they came on the market, by learning to use the council's online system.

…It’s a learning curve for them, a lot of them it’s the first time they’ve done anything like this. I like to celebrate the little wins. You put a bid in on a house, ok you didn’t get it, well now you can do that [put in a bid] so you’re in a better place than you were three weeks ago.

They saw this as part of teaching applicants to internalise a success‐based mindset, in which hard work, not chance, was understood as the mechanism through which the ‘good life’, in the form of secure accommodation, was realised. Failure was represented as a critical teaching point, where applicants were at risk of disengagement due to losing belief in the system. As one decision‐maker put it:

I’ve got to keep them going at that point because that’s when we can lose them, and that’s about saying, this is all part of it, you’ve got to take the rough with the smooth…it’s keeping going.

Some workers also used failure‐preparation to build resilience, through strategies to increase success in the longer term (Joseph, 2013). They individualised housing inaccessibility caused by structural shortage of affordable housing, and instead responsibilised applicants for developing skills to enable them to obtain housing. Applicants were discouraged from discussing difficulties; *‘complaining and negativity’*, as one hostel worker characterised it, was seen as compromising applicants' ability to plan for an improved future – *‘it just gets them stuck in that negative cycle and then I find you just can't move them on. You can't lift their eyes above the pavement so to speak.’* Another worker, a decision‐maker in a city centre office, explained how she dismissed realistic apprehension, and recognition of obstacles, framing them as a failure of personal willingness to investment in the future (Ahmed, 2010).

I say to them, don’t tell me no no no. I don’t want to hear it. What do you know that you didn’t know last week? Right then what are you going to do next? Where are you going to look, who are you going to call. Keeping them positive, keeping their eyes up, know what I’m saying.

Maintenance also demands that struggle is acknowledged, enabling people to properly prepare for it and so see it as normal. This included an affective element, including strategic honesty. To deny applicants' lived experience risked their own credibility, reducing their ability to affectively motivate applicants. Workers tended to see the system as fundamentally inequitable, but were motivated to help applicants to survive, and saw helping them sustain themselves despite set backs as an ethical way to do so (see England, 2023; England and Henley, 2024, 2024). As one hostel worker explained, *It's a bloody nightmare and I'm honest with them ‐ they'll hear more nos than yesses'.* One outreach worker explained how he tried to balance the reality of a competitive, exclusionary housing market with authentic, nurturing and motivating client connection

It’s a tightrope. You’ve got to keep them going. You can’t let them wallow. Same time, you’ve got to be real with them.

The difficulties faced by applicants increased the importance, for workers, of strategies to sustain them over a protracted, indefinite period of homelessness. This reflects Joseph's (2013) proposal that neoliberal interventions demand not only that citizens become *empowered* – able to manage their own lives in the increasing absence of state support‐ but *resilient*‐ able to anticipate and prepare for future setbacks. It was often characterised in terms of enabling personal growth – as one decision‐maker put it, *‘giving them empowerment’.* A hostel worker explained that she tried to increase applicants' capacity to withstand repeated disappointment and challenges by framing the private rented sector as both unfair and immutable. She proposed that this was necessary if they were to have any realistic prospect of success.

You can promise the world…there’s no point denying…what they’re facing, they’re facing. If I were to be here saying oh yeah all you got to do is pick up the phone, look on Rightmove. I’d lose them. It’s about keeping going, learning to pacing yourself…it’s not [going to] happen overnight….It’s about becoming someone who can do that, you know…

Workers were then highly concerned with finding strategies to maintain applicant engagement over a protracted period of repeated setbacks, offering applicants a workable and believable strategy to maintain their belief in the ultimate *necessity*, if not morality, of ongoing engagement with the neoliberal paternalistic incursion into the homelessness system.

## 3 CONCLUSION

Frontline workers provide a complex and liminal service in which anger and frustration is acknowledged, reframed and redirected: a crisis heterotopia in which change itself is foreclosed. Exploring the logics deployed by frontline workers to uphold the system, even in the context of their own misgivings, exposes the symbolic violence of neoliberal welfare governance as a systematic and strategic approach (Foucault, 1979), sustained through distributed, mundane micro‐interactions designed to incubate responsibilisation for structural inequality within marginalised subjects (Foucault, 2020). Drawing upon a Critical Discourse Analysis of interviews with workers in the statutory Welsh homelessness system, this paper has explored how frontline workers carefully curate uncertainty, hope and empowerment strategies to engender and maintain acceptance of individualised responsibilisation.

I have explored how this was planned and deliberate, arising from a deep conception of psychological reformulation as an urgent, responsible, act of care rendered necessary by a perception that homeless applicants had been abandoned by the state. I argue that workers systematically create and sustain optimism in their clients as a mechanism to enable them to survive within an increasingly hostile housing system., as part of a deliberate, if reluctant, strategy aimed at cultivating empowered, ‘ethical’ welfare selfhood against a backdrop of citizen abandonment. I developed and demonstrated a three‐part typology to enable Berlant's cruel optimism to be used to explore technologies of governance. *Disruption* instigates a desire for change by positioning the present situation as unsustainable and potentially dangerous. Here, workers were shown to be instrumental in communicating that state welfare provision, specifically housing assistance for those experiencing homelessness, had become precarious, scarce and unsustainable. Despite expressing unease at the change in private, they also saw cultivating this awareness in individual applicants as a necessary step to protect them from the effects of a system‐wide state abandonment. *Re‐orientation* harnesses imagination to engender attachment to new possibilities, while encouraging subjects to see correct desire, when coupled with appropriately compliant behaviour, as the route to success. Workers engaged applicants in structured programmes of imaginative dreaming, providing a motivation for change. Finally, the *maintenance* phase is critical to preventing people from giving up when they inevitably fail. It is essential to retain engagement with the wider neoliberal project (Lynch et al., 2020; J. Tronto, 2017). Workers felt considerable ambivalence about renarrativising failure; at this stage, they themselves often began to question the morality of their roles and started to see optimism as potentially cruel. Yet in most cases they continued because it was felt to be a kinder option than letting applicants fail.

This extended, three stage, approach to cultivate empowered, resilience strategies in applicants, engendering ‘ethical’ forms of welfare citizenship (Rose & Miller, 2010) was deployed not because workers themselves believed these logics but because, appraising the withdrawn state from an insider standpoint, they feared for applicants who would not or could not act as neoliberal citizens. Care, rather than revanchism, therefore drove cultivation of neoliberal citizenship. They destabilised, cultivated and maintained neoliberal logics among homeless applicants because they believed it was the kindest and most productive way for them to help.

It is also important to recognise that both those experiencing homelessness, and those administering state bureaucratic systems, are both complex and resistive. Cruel Optimism operates by engendering desire to comply, through instilling a hopeful belief that compliance will produce a tangible reward. However, as Foucault observes, power is necessarily uneven and partial, operating *through* individuals, and so shaped and amplified, but also limited by, prior experience, social position, relationality, and expectations (Hall, 2001). There is evidence that workers perceive applicants within the Welsh homelessness system differently. For instance, lone parents, and (to a lesser extent) families are especially likely to be understood in moralistic terms and seen as neoliberal failures in need of re‐education (England, 2024, 2024). Further work might consider how these differences in subjectivity give rise to different experiences of Cruel Optimism.

Neoliberalism relies upon pervasive, encompassing and enrolling logics, incorporating and reframing resistance as compliance (Lynch et al., 2021; J. Tronto, 2017). On one level, workers can be seen to operate to reinforce neoliberalism, by taking decisions to normalise it *to* help their clients. Responsibilising poor and marginalised individuals by reframing the impacts of oppression as surmountable through becoming better neoliberal individuals can be understood as an especially effective method to advance the forms of intrusive psychological poverty governance associated with neoliberal paternalism (Berlant, 2011; Lynch et al., 2020; Skeggs, 2014).

This paper contributes to understanding how frontline workers themselves understand and instigate change, by exploring how affective governance can be perceived by workers not as a punitive or manipulative act, but rather as positive and optimistic, enabling transfer of knowledge. It shows that frontline workers use strategies derived from elements of Cruel Optimism not because they wish to instigate harm, but because they want to protect applicants – because they care. In a withdrawn, neoliberalised, system, their options become extremely limited, and, as I demonstrate in this paper, hope – even at the expense of truth – can become critical to psychological wellbeing.

## CONFLICT OF INTEREST STATEMENT

The author declares none.

## Data Availability

Research data are not shared.
